# Improving treatment outcomes in Ghana with agent-based model for diabetes patients’ self-management behaviours

**DOI:** 10.1007/s10729-026-09772-8

**Published:** 2026-07-25

**Authors:** Eunice Twumwaa Tagoe, Justice Nonvignon, Robert Van Der Meer, Itamar Megiddo

**Affiliations:** 1 Population Health Sciences Institute, Newcastle University, Newcastle Upon Tyne, UK; 2 School of Public Health, University of Ghana, Accra, Ghana; 3 Department of Management Science, University of Strathclyde, Glasgow, UK

**Keywords:** Diabetes, Type 2 diabetes, Agent-based modelling, Pattern-oriented modelling, Multiple validation levels, Self-management behaviour, Medicine adherence

## Abstract

Despite universal health coverage policies, diabetes management in resource-constrained settings remains challenging due to complex interactions between patient behaviours and systemic barriers. This study combines health behaviour theories with healthcare system constraints in an agent-based model to evaluate the influence of policy scenarios on type 2 diabetes patients’ self-management behaviours, blood glucose control, medication adherence, and hospital admissions within Ghana’s public health system. Using pattern-oriented modelling with multiple validation levels, the model integrates empirical evidence from clinical data, service provider expertise, and behavioural research to simulate how patients’ daily decisions about taking medicines, following lifestyle recommendations, and seeking healthcare interact with system-level factors like medicine availability and insurance coverage. We evaluate three public health scenarios: expanding insurance coverage, increasing medicine availability, and implementing a sugar-sweetened beverage tax. Our analysis indicates that a sustained 10% annual increase in insurance coverage over five years yields substantially greater improvements in blood glucose control and reduces hospital admissions compared to alternative scenarios. By integrating behavioural theories with system constraints to simulate patient decision-making, this modelling approach reveals how affordability and availability barriers interact to influence self-management behaviours and treatment outcomes in underserved populations. Our findings have implications for the operation of Ghana's national insurance scheme and diabetes service planning and delivery within the public health sector.

**Supplementary Information:**

The online version contains supplementary material available at https://doi.org/10.1007/s10729-026-09772-8.

## Highlights

- The study integrates behavioural theories with healthcare system constraints in an agent-based model to evaluate policy scenarios in resource-limited settings, revealing how availability and affordability barriers interact to influence diabetes treatment outcomes.
- The study demonstrates significant disparities in treatment outcomes between rural/urban and insured/uninsured populations, with financial barriers having a greater impact than medicine availability on self-management behaviours.
- The model provides quantitative evidence suggesting that expanding insurance coverage yields greater improvements in blood glucose control than alternative scenarios by reducing financial barriers.

## Introduction

Despite Ghana's national health insurance coverage for diabetes care, many patients struggle to achieve adequate blood glucose control due to challenges with medication access, affordability, and adherence [1, 2]. These barriers particularly affect patients in resource-constrained settings, where medicine stockouts are common, and the ability to pay remains a significant obstacle to consistent treatment [3]. Understanding how patients' self-management behaviours interact with health system constraints is crucial for identifying effective policy scenarios to improve diabetes outcomes in these underserved populations.

This study examines how patient self-management behaviour influences blood glucose control, medication adherence, and hospital admissions within Ghana's public health system. Using health behaviour theories and agent-based modelling, we evaluate three policy scenarios: expanding insurance coverage, increasing medicine availability, and implementing a sugar-sweetened beverage tax. Our analysis focuses on type 2 diabetes (T2DM) patients receiving care in public facilities, where resource limitations and systemic barriers often compromise treatment effectiveness.

The burden of diabetes in Ghana's public health system continues to grow, with prevalence among the working-class population (20—79 years) in 2021 estimated to be roughly 320 (145.2 – 538.5) thousand and expected to increase by about 134% by 2045 [4]. While diabetes services are covered under Ghana's National Health Insurance Scheme (NHIS), inadequate medicine availability, limited insurance coverage, and poor adherence to treatment regimens contribute to suboptimal outcomes. Less than 50% of diabetes patients achieve good glycemic control, with rates even lower among uninsured and rural populations [5]. These challenges are compounded by rising obesity rates and limited resources for chronic disease management in the public sector [3, 6].

Patients’ self-management behaviour plays a critical role in diabetes control, yet simulation modelling approaches for health system interventions often fail to capture the complex interactions between individual behaviours and systemic constraints. For instance, uninsured patients may miss hospital appointments due to affordability issues, and they may not receive prescriptions to buy medicines, leading to nonadherence, poor blood glucose control and possibly admissions. Traditional Markov cohort models cannot represent the network of factors influencing treatment adherence [7]. Agent-based models (ABMs) can model the interaction of individual-level patients' behaviours and system-level constraints in generating population-level glycemic control outcomes [8, 9]. Modelling approaches that cannot account for these interactions have limited our ability to evaluate self-management behaviour and public health scenarios for T2DM among underserved populations [9].

To address these challenges, we develop an ABM that integrates health behaviour theories with national treatment guidelines, service providers’ experiences and published data to simulate patient self-management behaviour and treatment outcomes. Our model represents key barriers faced by underserved populations, including medicine availability, insurance coverage, and lifestyle factors affecting treatment adherence. This approach allows us to evaluate how policy scenarios might improve glycemic control while accounting for individual behaviours and system-level constraints.

The rest of the paper is organised as follows: Section 2 presents literature on self-management behaviour and modelling approaches. The methodology used in developing and validating patients’ behaviours is presented in Section 3. The ABM process, policy scenarios, experiments, and outcomes assessed are also presented in Section 3. Section 4 presents the results from our model validation and policy scenario experiments. Section 5 discusses the implications for improving diabetes care delivery for underserved populations in Ghana, with conclusions and limitations in Section 6.

## Literature review

### Self-management behaviours and treatment outcomes

Even the most well-designed interventions can fail if patients do not adhere to treatment regimens or adopt recommended lifestyle changes. Therefore, understanding patient self-management behaviour provides valuable insights for modelling and is crucial for developing and implementing successful diabetes control interventions. The following section delves into the complexities of patient self-management behaviour, examining the theories explaining health-related actions and the multifaceted factors influencing treatment adherence and outcomes.

Although good adherence and a healthy lifestyle improve blood glucose control and reduce health service utilisation [10, 11], many patients struggle to maintain recommended behaviours. The WHO asserts that healthy self-management behaviour may have a far bigger effect on population health than any change to a particular medical intervention because it reduces risk factors and prevents adverse health outcomes [11]. About one-third of diabetes patients globally are not adherent to medication, with depression and cost being major determinants [12]. In Ghana specifically, the proportion of good medicine adherence among T2DM patients ranges from 38.5% to 84.5% [2, 13, 14].

Lifestyle challenges are particularly evident in Ghana, where few people eat healthily and regularly exercise [15, 16]. Obesity prevalence almost doubled while overweight prevalence tripled between 1993 and 2014 in Ghana [17], indicating increased unhealthy diet consumption and decreased physical activity. Over 40% of T2DM patients in Ghana are overweight/obese diabetes, increasing their risk of complications and poor blood glucose control [5, 6].

Evidence suggests that the challenges to self-management behaviour are interrelated, with particularly severe impacts for underserved populations. Studies have shown that poverty affects not just medicine access but healthcare engagement—even with insurance, co-payments and insurance renewal costs influence patients to abandon orthodox medicines for traditional alternatives and faith healers [18, 19]. This financial barrier appears especially acute in rural and low-income urban areas, where studies document lower engagement with health services and insurance coverage [20, 21]. The economic constraints interact with healthcare delivery challenges, as few trained service providers and suboptimal referral and appointment scheduling systems further contribute to nonadherence and loss of follow-up [22]. Rural populations face additional challenges from the geographic distribution of healthcare resources, while the urban poor encounter barriers despite physical proximity to facilities [3]. While these individual barriers are well-documented, their combined effects on self-management behaviours and treatment outcomes, particularly in resource-constrained settings like Ghana, are less well understood.

### Health behaviour theories for explaining self-management behaviours

The Health Belief Model (HBM) and the Theory of Planned Behaviour (TPB) provide complementary frameworks for understanding diabetes self-management behaviours, considering multiple layers of influence–individuals, social, economic, and system-level. While HBM emphasises the role of individual perceptions–such as perceived susceptibility, severity, benefits, barriers and self-efficacy–in influencing behaviours [23, 24], TPB incorporates social influence in shaping behaviours [25]. TPB suggests that attitudes and subjective norms about how others expect one to behave, which, alongside behavioural control in HBM, determine behaviour. Together, these theories provide a multidimensional insight into self-management behaviours, integrating personal beliefs with social and structural contexts—an approach particularly relevant in resource-limited settings where patients face multiple barriers to care.

Research has applied these theoretical frameworks to explain and predict various health behaviours, including medication adherence, diet, and physical activity. Studies typically identify targeted behaviours, classify their predictors under theoretical constructs and use surveys and statistical methods to investigate the relationship between constructs and self-reported behaviours [26–28]. In chronic disease management specifically, researchers have used the theories to understand treatment adherence and design interventions [29–31]. In Ghana, Mogre et al. [31] applied TPB to understand T2DM self-management barriers, highlighting the importance of considering resource constraints when applying these frameworks. Evidence suggests that community norms and social influence significantly impact diet and exercise behaviour [32, 33], while barriers such as medicine affordability, stockouts, and prescription requirements influence medicine adherence [34, 35]. Perceived severity, symptoms of ill health, and perceived susceptibility have been associated with health-seeking behaviours [36], further supporting the applicability of TPB and HBM for conceptualising diabetes self-management [33, 37, 38].

For computational modelling, particularly in resource-constrained settings, HBM and TPB offer unique advantages beyond their explanatory power. By mapping theoretical constructs to specific behavioural rules in an ABM, we can simulate how patients navigate healthcare system constraints based on established behavioural principles, rather than relying solely on statistical associations that may not capture causal mechanisms [39, 40]. The approach also provides a structured framework for overcoming a lack of longitudinal data on behavioural processes, such as how perceived barriers affect medication adherence [41, 42]. By combining theoretical frameworks with limited available data, we can strengthen confidence in the models’ validity.

### Modelling approaches for health behaviours

ABM offers distinct advantages over traditional modelling approaches. While statistical approaches help identify relationships between behavioural factors, they cannot capture the dynamic interactions between patient behaviours and healthcare system constraints. Markov cohort models cannot represent heterogeneity in patient characteristics and behaviours. ABMs are particularly suited for examining individual decision-making processes and their interactions with environmental constraints, which is particularly important for understanding how barriers affect different patient groups and offers potential for integrating behavioural theories with healthcare system dynamics.

Our approach builds on previous applications of ABM to health systems but addresses an important gap by integrating both behavioural theories and system constraints to understand chronic disease management in resource-limited settings. Most ABMs that integrate health behaviour theories are for high-resource settings and focused on emergency or acute care and health insurance schemes [43, 44]. Luo et al. [45] modelled individual diet, physical activity, and smoking behaviours in China using social influence theory, where agents adjusted their behaviour based on interactions with neighbours. Specifically, individuals in the model adjust their diet, physical activity and smoking behaviours based on the behaviours of their social contacts. In Ghana, ABM have been applied to study 1) cholera transmission, adaptive and protective behaviours [46, 47], 2) urbanisation [48] and 3) land, water and agriculture [49, 50]. Abdulkareem et al. developed an ABM on cholera in Ghana and incorporated Protection Motivation Theory to represent how individuals assess health risks and choose coping strategies [46]. The authors used the theory to inform modelling how individuals at different infectious states interacted with other individuals and their environment.

For integrating behavioural theories into ABMs, Schlüter et al. [39] provide a framework that explicitly represents decision-making processes at the individual level while accounting for social and biophysical environmental influences. This approach is particularly relevant to modelling chronic disease self-management behaviours, where patient decisions occur within constraining healthcare environments.

## Methodology

This section describes the ABM model description, data and parameterisation, and confidence building. We discuss the scenarios modelled, experiments and observed outcomes.

### ABM model overview and assumptions

Our ABM model simulates how T2DM patients’ self-management behaviours interact with healthcare system constraints to influence treatment outcomes in Ghana’s public health system. Through this model, we evaluate how policy scenarios targeting system-level constraints and behaviour affect blood glucose control.

**Fig. 1:**
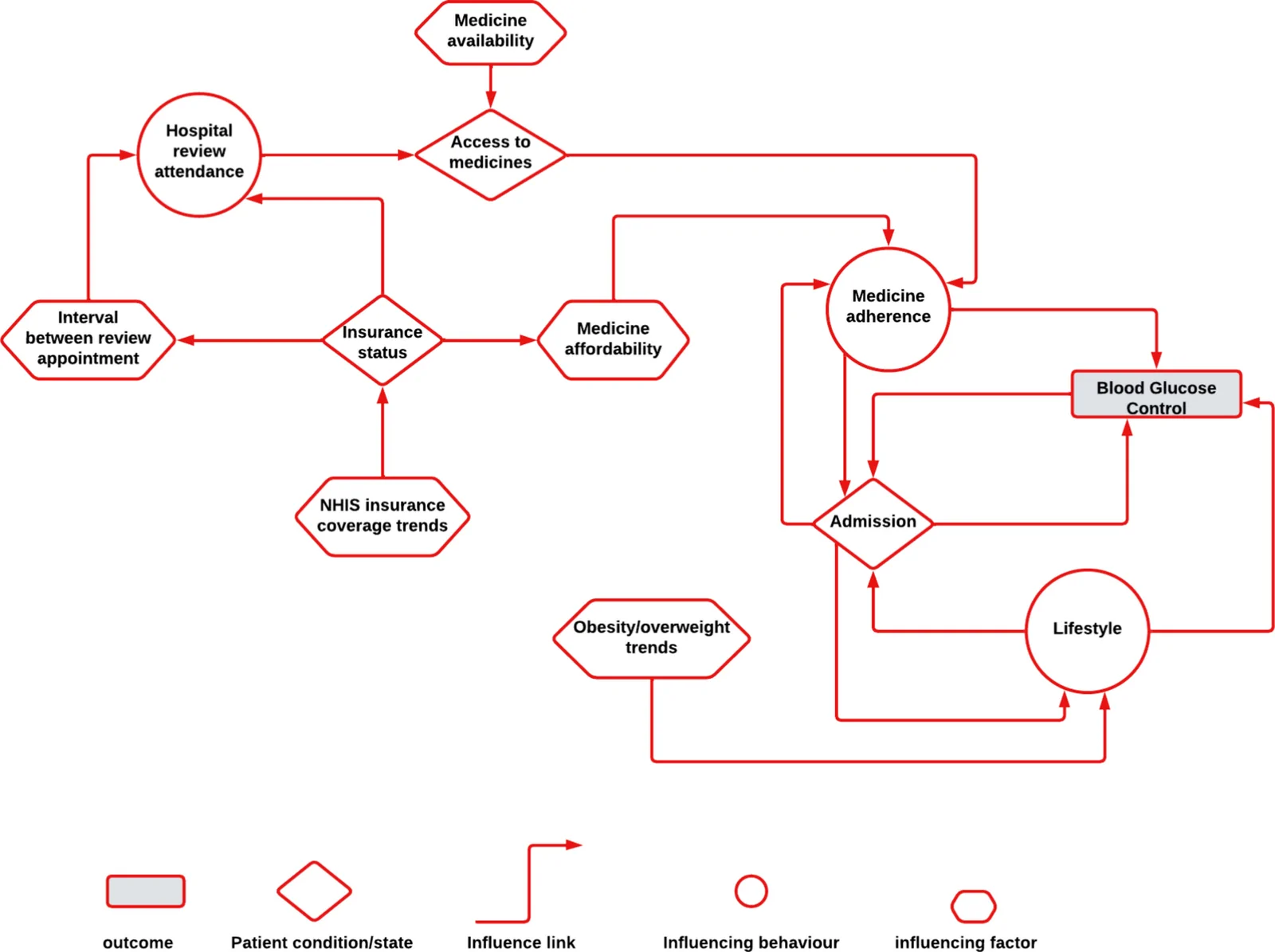
Relationships between model variables and blood glucose outcome

The model consists of a cohort of patient agents who make decisions about medication adherence, lifestyle choices, and healthcare seeking within a constrained healthcare environment. The cohort ages over time without new patients entering the model. This approach allows us to track how patients’ behaviours and outcomes evolve as they age and interact with the healthcare system. Figure 1 illustrates how system constraints and patient behaviours interact to influence blood glucose control. Detailed model description and assumptions are published here: https://doi.org/10.15129/e0d78b33-152f-44dc-9d8e-07886db72288.

Insurance status affects both the frequency of scheduled appointments and medicine affordability, while medicine availability in rural/urban areas directly impacts access. Patients must navigate these constraints while managing regular review appointments, which provide opportunities for prescriptions and monitoring. These factors determine medication adherence; they also combine with lifestyle choices to influence blood glucose control and subsequent healthcare interactions.

**Fig. 2:**
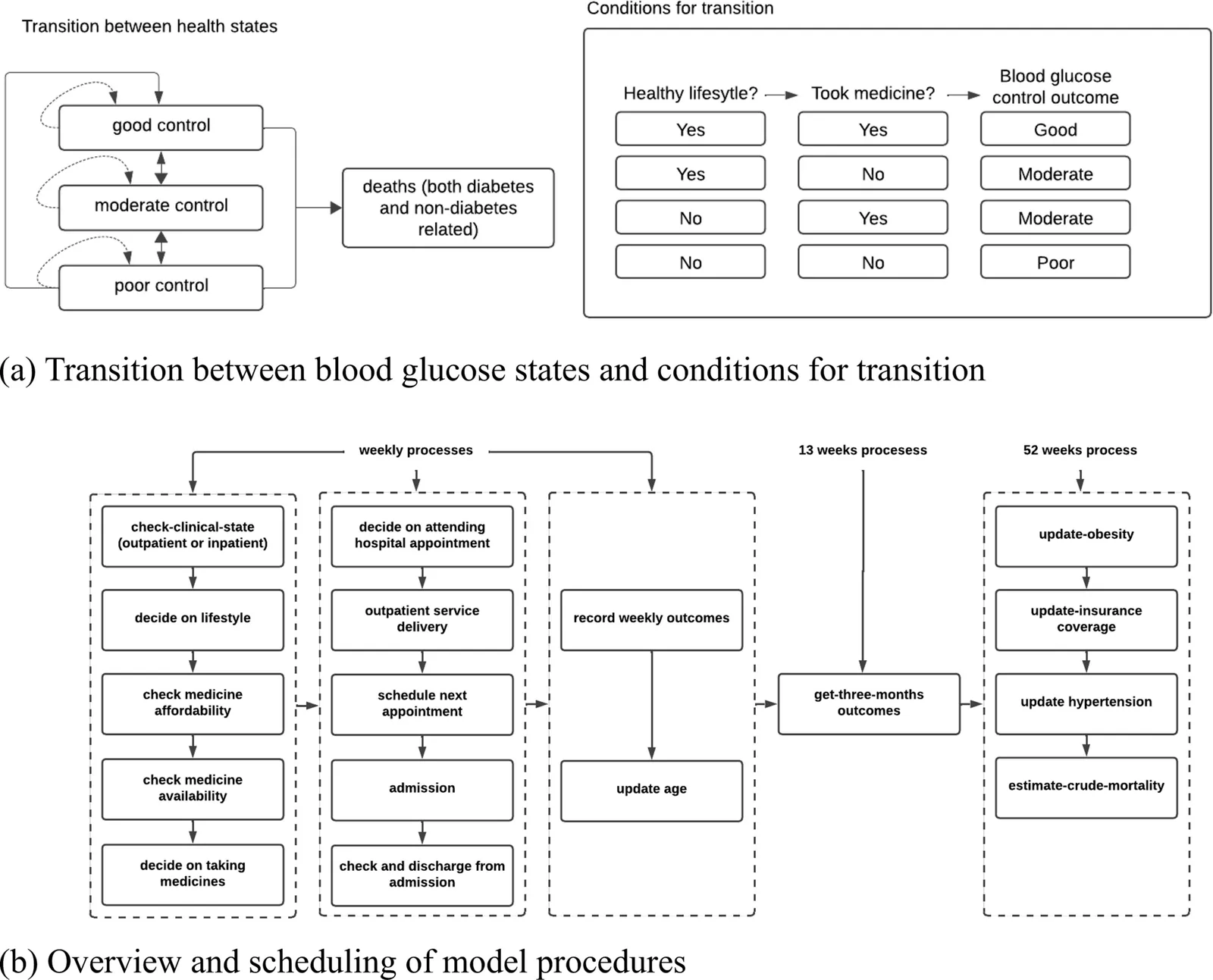
Transition between diabetes states and scheduling of model processes. (**a**) Patients transition between blood glucose control states. Clinical intervention can move patients directly from poor to good control; without intervention, changes occur incrementally. (**b**) Weekly sequence of patient decisions and healthcare interactions, with updates to individual outcomes every 13 weeks and population-level factors

The model processes and transitions are shown in Fig. 2. Patient agents can be in different blood glucose control states, which evolve based on their behaviours and healthcare interactions (Fig. 2a). The model timestep is one week; each week, patients follow a structured sequence of decisions and actions as they engage with the healthcare system (Fig. 2b). The decision rules and their basis in theory are explained further in the following section.

The model’s assumptions are grounded in HBM, TPB, and clinicians’ opinions (see Table 1 and Appendix 2), as explained in Section 3.2. Several key simplifying assumptions shape the model’s structure. First, we use obesity and overweight prevalence as a proxy for aggregate lifestyle behaviours, including diet and physical activity; the implications of this simplification are examined through structural sensitivity analysis (Section. 3.4) and discussed in the study limitations. Second, patients’ lifestyle behaviours are assumed to be primarily influenced by close social contacts, patterned by rural and urban settings. Third, inpatients are assumed to comply with prescribed medicines and lifestyle recommendations under clinicians’ supervision, with admission lasting 1–2 weeks. Fourth, medicine adherence is determined by a sequence of practical constraints: having a valid prescription (linked to appointment attendance), medicine availability, and affordability (determined by insurance coverage and wealth). Patients who miss outpatient appointments do not obtain prescriptions and therefore do not take medicines. Finally, comorbid patients with poorly controlled glucose experience symptoms and seek care without a scheduled appointment. Each assumption is operationalised through the decision rules described in Section. 3.2.

### Decision rules and transitions

#### Patients

Patient decision-making integrates constructs from the HBM and TPB to represent self-management behaviours. Table 1 shows how these theoretical constructs are mapped to specific behaviour rules and provides justification for their use. The key behaviour mechanisms are implemented as described below, with a detailed description provided in the submodel section of the model description published here: https://doi.org/10.15129/e0d78b33-152f-44dc-9d8e-07886db72288. Supplementary File 3 shows how the implemented model procedures were verified against the conceptual model to ensure accuracy.

**Table 1:** HBM and TPB application to self-management behaviours in T2DM patients

Behaviour	HBM Tenets	TPB Tenets	Application in the ABM model	Operationalisation	Justification
Lifestyle (diet and exercise)	N/A	Subjective norms	Community norms influence lifestyle choices (outpatients); provider guidance influences inpatient behaviour	Outpatients’ lifestyle choices stochastically follow local community trends. Inpatients have healthy lifestyle	A large body of literature show that community norms, social desirability, influence, and integration strongly affect diet and exercise behaviours [31, 32] and TPB is commonly used to explain choices [37, 51]
Medication intake (with prescription)	Perceived barriers	Actual behavioural control	Medicine intake is constrained by affordability (insurance/wealth status) and local availability	Do not take medicine if: 1. cannot afford 2. medicine not available	HBM is well-suited for modelling barriers to medication adherence, as it specifically examines how perceived barriers influence health behaviours [27]. TPB’s construct of actual behavioural control effectively captures how external constraints hinder medication-taking despite intentions [51]. Studies in Ghana confirm affordability and availability constrains exist [52, 53]
Medication intake (no prescription)	Perceived barriers	Actual behavioural control	Prescriptions requirement as access barrier; missed appointments lead to prescription expiry	Do not take medicines if no prescription due to missed appointments	
Review appointment attendance	Perceived severity and cue to action	Attitude	Symptom and perception influence appointment attendances	Probability of attendance decreases when asymptomatic	HBM's constructs of perceived severity and cues to action are particularly suited for modelling appointment attendance as they explain why symptom perception triggers healthcare-seeking [27]. Research shows these constructs predict appointment adherence in chronic disease management [54], with theoretical validity demonstrated in multiple Ghana-based studies [55]
Seeking care in acute complication state	Perceived severity and susceptibility Perceived benefit	Attitude	Poor glucose control and symptoms trigger care-seeking	Seek immediate care when experiencing poor blood glucose symptoms	
Inpatients’ behaviour	Perceived severity and susceptibility	Subjective norms	Direct provider supervision influences adherence	Follow prescribed medication and lifestyle regimes under supervision	TPB’s subjective norms construct illustrates how healthcare providers impact patient behaviour in supervised settings [27]. HBM’s perceived susceptibility and severity constructs clarify why the hospital environment amplifies patients’ sense of disease threat [54], making both theories well-suited for modelling inpatient adherence behaviour
Discharge patients’ behaviour	Perceived severity and susceptibility Perceived benefit	Attitude	Recent admission experience influences post-discharge behaviour	Continue recommended behaviors from hospital stay for a period, subject to access constraints and until the next outpatient appointment	HBM's perceived susceptibility construct explains why recently-discharged patients maintain behaviours due to heightened illness awareness [27]. TPB's attitude construct provides theoretical foundation for how recent admission experiences modify patients' evaluations of treatment benefits [51], creating a temporary change in adherence behaviour

*HBM* Health Belief Model, *TPB* Theory of Planned Behaviour, N/A means there is no construct from the Health Belief Model that we combined with the Theory of Planned Behaviour to conceptualise a behaviour

##### Lifestyle decisions

For outpatients, lifestyle choices about diet and exercise reflect social influences through TPB’s subjective norms construct. This is operationalised by modelling patients’ lifestyle choices as influenced by community trends, using stochastic processes that are driven by local prevalence in obesity and overweight. Patients follow clinical guidelines during hospital stays, with the healthcare environment becoming the normative influence (see Table 1).

##### Medication intake

Medication adherence decisions combine multiple theoretical constructs. The HBM’s perceived barriers and TPB’s actual behavioural control are represented through a sequence of practical constraints: patients must have both a valid prescription and the ability to pay for medicines before medicine availability is considered. As shown in Fig. 2b, patients first check medicine affordability—those with insurance or in middle to high-wealth quintiles are assumed able to afford medicines when available. If medicines are unavailable or patients cannot afford them, this represents an actual behavioural control barrier preventing medication taking, regardless of intention.

##### Healthcare seeking

Health-seeking behaviour combines constructs from HBM and TPB (as outlined in Table 1). Patients’ decisions to attend scheduled appointments to seek care when experiencing symptoms are modelled based on these theoretical frameworks. Symptomatic patients experiencing poor blood glucose control seek immediate care, even without scheduled appointments, representing HBM’s cue to action and perceived benefits constructs. Conversely, asymptomatic patients may skip scheduled appointments due to low perceived threat, demonstrating how perceived severity influences healthcare engagement. The model incorporates these theoretical constructs by adjusting attendance probability based on symptoms and appointment timing.

##### Health state transitions

Figure 2a (left side) shows how patients transition between blood glucose states (good, moderate, and poor). These transitions are directly influenced by patients’ lifestyle choices and medication adherence behaviours, representing the outcomes of the decision process explained in previous sections (see Supplementary File 1 for details). With clinical intervention during hospital admissions, patients can transition directly from poor to good control; otherwise, transitions occur incrementally between adjacent states.

The interaction between lifestyle and medication adherence determines these transitions: patients maintaining both a healthy lifestyle and good medication adherence (^3^80% of prescribed doses) achieve good control; those with an unhealthy lifestyle and poor adherence (< 50%) develop poor control; while mixed behaviours (good in one domain but poor in another) result in moderate control. These transition rules operationalise the theoretical constructs by connecting behavioural decisions to physiological outcomes. These operational definitions are supported by evidence linking specific adherence thresholds and lifestyle behaviours to clinical outcomes [51, 56]. The model also incorporates background mortality-based age-specific rates, allowing patients to exit the simulation through death.

#### Healthcare delivery

Healthcare delivery rules, in the model, reflect current clinical practice in Ghana’s public health system. These rules were developed using national diabetes treatment guidelines [36] and insights from clinicians (see Supplementary File 1 for details).

##### Clinical service rules

Healthcare facilities provide both outpatient and inpatient care. During outpatient appointments, providers assess blood glucose control and issue prescriptions valid until the next scheduled appointment. All patients receive a standard review appointment interval of 13 weeks if their blood glucose is controlled. However, patients presenting with poor blood glucose control, but no comorbidities, receive earlier follow-up appointments after their glucose is brought to controlled levels. We do not model delayed outpatient appointments due to increases in inpatient cases and fixed service provider capacity, as providers indicated that such conditions are rare.

##### Admissions criteria and management

Patients with poor blood glucose control who also have comorbidities (such as hypertension) experience symptoms of ill health, seek care, and are admitted for inpatient care due to the high risk of complications and need for supervised management. Hospital stays are limited to a maximum of two weeks, during which patients receive supervised medication administration and lifestyle guidance. After controlling blood glucose, patients are discharged with prescriptions and follow-up outpatient appointments.

##### Medicine dispensing

Access to diabetes medicine requires a valid prescription from a healthcare provider. When patients attend appointments, they receive prescriptions that remain valid until their next scheduled visit (see Supplementary File 1 for details). Medicine dispensing is subject to both availability in the facility and patients’ ability to pay. For insured patients, medicines are provided without out-of-pocket payments. Uninsured patients must pay the full cost.

### Data sources and parameters

The model parameters, key ones provided in Table 2, were derived from multiple sources (detailed parameters in model description published online: https://doi.org/10.15129/e0d78b33-152f-44dc-9d8e-07886db72288).

Population-level parameters describing sociodemographic characteristics (age, insurance status, wealth quintiles) and disease-specific factors, including comorbidity rates (proxied through hypertension prevalence in this population), were based on Ghana Demographic and Health Survey data, Ministry of Health Report, Ghana Multiple Indicator Cluster Survey and Ghana-specific diabetes studies [15, 17, 57, 58, 62], details in the published model description. Parameters governing patient behaviours were derived from HBM, TPB and diabetes studies [13, 63]. We used reported rates of medication adherence and appointments to calibrate behavioural probabilities [1, 5, 64, 65]. Lifestyle parameters were based on population-level obesity and overweight prevalence trends [17], which are proxies for diet and physical activity behaviours. The choice of proxies is justified by evidence showing that diet and physical activity behaviours closely align with trends in obesity and overweight prevalence [10, 59]. Environmental parameters include urban/rural differences in medicine availability and healthcare access, derived from a national pharmaceutical availability study in Ghana [62].

Healthcare delivery parameters were informed by Ghana’s diabetes treatment guidelines and consultation with service providers. We established and confirmed parameters for appointment intervals, admission duration, and service delivery protocols through semi-structured interviews and informal consultations. Medicine availability parameters were derived from literature [62] and consultation with diabetes service providers in Ghana. Summaries of the patterns and findings from our engagement with service providers are in Supplementary File 1.

**Table 2:** Key model parameters

Parameter name	Meaning and rationale	Base case value	Range (used in sensitivity analysis)	Source Type	Source
***Population-level***					
UrbanProp	The proportion of the population living in urban areas. Used at initialisation to populate patients' residence	0.52	0.50 – 0.58	Survey data	Ghana Statistical Services [57]
MedUrban	The percentage of times DM medicines are available to patients in urban communities. Used in setting med-in-com, which indicates if the patient found medicines. Details in check-meds sub model in model description	0.8	triangular distribution (min = 0.6 mode = 0.8 max = 1)	Literature	Assumed based on Masters et al. [58]
MedRural	The percentage of times DM medicines are available to patients in rural communities. Used in setting **med-in-com,** which indicates if the patient found medicines. Details in **check-meds** sub model	0.7	triangular distribution (min = 0.4 mode = 0.7 max = 1)	Literature	Assumed based on Masters et al. [58]
InsuranceCoverage	The proportion of the cohort actively insured under the NHIS. Used at initialisation in the **set-insurance** procedure and the **update-insurance** submodel	Estimated from an S-shaped distribution with equation and parameters (details in submodel section in the model description)		National Health Report	Ministry of Health [59]
HypertensionPrev	The annual prevalence of hypertension and overweight (used as a proxy for comorbidity). Used to increase the prevalence of comorbidity among patients)	Estimated from an S-shaped distribution with equation and parameters (details in submodel section in the model description)		Literature	Opoku et al. [60]; Adeloye and Basquill [61]
PDiet	The prevalence of obesity and overweight (used as a proxy for diet and exercise behaviour). Used to increase the prevalence of poor diet and exercise behaviours among patients)	Estimated from an S-shaped distribution (details in update-obesity sub model section in the model description)		Literature	Amugsi et al. [17]
ruralProDiet	The proportion of the national prevalence of obesity and overweight in rural communities	0.4			Yussif et al. [6]
urbanProDiet	The proportion of the national prevalence of obesity and overweight in urban communities	0.6			Yussif et al. [6]
***Patient-level parameters***					
age	Age of patients. used at initialisation to populate patients' age	Mean = 59 Standard deviation = 12 Min = 27 Max = 88		Literature	Osei-Yeboah et al. [14]
DiabetesDuration	The proportion of patients who have had DM for a given number of years. used at initialisation to populate patients' *duration* state variable in the **set-duration** sub model of the model description	1–5 years = 46.7% 6–10 years = 29.3% > 10 years = 24.0%		Literature	Osei-Yeboah et al. [14]
***Service delivery parameters***					
ReviewInterval	The maximum number of weeks between outpatient appointments is used in the **schedule-appointment** procedure within the **treat** submodel (in the model description) to populate patient appointment weeks	13	uniform distribution min = 8 max = 18	Expert consultation	Estimated from clinicians' opinion
AttendanceProp	The proportion of patients who attend outpatient appointments at scheduled times. Used in the **attend-review** sub model	0.6923	uniform distribution min = 0.38 max = 1.00	Literature	Amaltinga [13]

A comprehensive list of model parameters is presented in the model description published here: https://doi.org/10.15129/e0d78b33-152f-44dc-9d8e-07886db72288

### Model fitting and confidence building

#### Population trend fitting

Our approach first required fitting temporal trends for key population characteristics as model inputs: insurance coverage patterns, lifestyle factors (proxied by obesity/overweight prevalence), and comorbidity patterns (proxied by hypertension prevalence). We used the Sigmoid function based on evidence that obesity and overweight, population-level insurance coverage, and hypertension prevalence tend to follow a sigmoidal distribution [66]:

$$\mathrm{P}(\mathrm{t})=\mathrm{A}*\mathrm{K}/(\mathrm{A}+(\mathrm{K}-\mathrm{A})*\mathrm{e}\mathrm{x}\mathrm{p}(-\mathrm{r}*\mathrm{t}))$$

where r is the shape parameter of the Sigmoid curve, P(t) is the prevalence of obesity and overweight at time t, A is the initial prevalence, and K is the upper limit prevalence.

The function parameters were calibrated to match observed data patterns from 21 years (1993–2014) national obesity and overweight prevalence trends [17], active NHIS coverage trends from 2019–2023 [60] and hypertension prevalence from 1990–2017 [67, 68]. Above 80% predictive accuracy was considered a good fit of the data. See the model description document for details.

#### Pattern-oriented modelling and testing

For evaluating policy scenarios, we need confidence that our model captures key mechanisms driving substantial changes in outcomes and can appropriately represent how different system components interact. Building on the population trend fitting, we adopted a pattern-oriented modelling approach, drawing from complex system modelling in ecology [69]. This approach, along with the use of various forms of data, is particularly valuable in contexts where empirical data may be limited for some aspects of the system, as it helps build confidence in model behaviour by examining multiple complementary patterns across different system levels, rather than relying on a single validation metric. By validating the model against patterns at population, individual, and process levels, we aim to capture the key mechanisms driving system behaviour while avoiding overfitting to specific data points.

We explored these patterns bottom-up to test whether the model could reproduce patterns across three complementary levels:

1. Population-level outcomes: Blood glucose control and medication adherence rates compared against empirical data from Ghana (an 18-month prospective study with 1226 patients conducted in 2017– 2018) and similar settings (LMICs studies conducted between 2009 and 2017). Model outputs were considered reliable if control rates fell within the reported 95% confidence interval of poor control and adherence in these studies.
2. Individual-level behavioural responses were examined through extreme scenario testing of insurance status, medicine availability, and lifestyle factors. Three physicians with 2 + years of experience participated in a structured validation process. First, providers were asked open-ended questions about behavioural factors and barriers before seeing model outputs. In subsequent meetings, they reviewed scenario outputs (100% insurance coverage, 100% medicine availability, 50% reduction in unhealthy lifestyle) and assessed whether model responses matched expected behavioural patterns. All providers confirmed the model captured realistic behavioural responses to these system constraints, particularly noting that affordability barriers had stronger effects than availability barriers.
3. Treatment processes: Basic patterns in medication adherence decisions assessed against empirical evidence [1, 65], with decisions on appointment attendance and service delivery rules examined through provider consultations to ensure representation of their experience and clinical practice (see Supplementary File 1 for details).

#### Sensitivity analysis

##### Structural sensitivity analysis

We examined structural uncertainty by testing three alternative formulations for blood glucose estimation. The rationale was to assess whether our simplified approach (based primarily on medication adherence and lifestyle, which providers have identified as the key components) adequately captures the complexity of blood glucose control, or whether additional factors significantly alter model predictions. We focused on blood glucose estimation because this is the primary outcome of interest and incorporates factors with theoretical and empirical uncertainty. The base case (Model 0) estimated control from lifestyle and medicine adherence only. Alternative formulations included: Model 1 using weekly instead of 13-week blood glucose outcomes (based on Ghana's Diabetes Treatment Guidelines recommendation for capillary testing); Model 2 adding comorbidity effects; and Model 3 adding diabetes duration effects (both based on provider identification of key factors beyond lifestyle and adherence). Table 3 shows how each model was implemented and compared.

**Table 3:** Structural sensitivity around blood glucose control estimation

Uncertainty	Factors included	Baseline	Structural Model Implementation
Model1	Lifestyle, medicine adherence	13-week lifestyle and medicine adherence combined to estimate control	Control estimated weekly based on lifestyle and medicine adherence, with each factor weighted equally. *
Model2	Lifestyle, medicine adherence and presence of comorbidity		13-week lifestyle, medicine adherence AND comorbidity, with absence of comorbidity modelled as protective.^a b^
Model3	Lifestyle, medicine adherence and diabetes duration		13-weeks lifestyle, medicine adherence, AND diabetes duration: if duration is less than 5–10 years (randomly selected from a uniform distribution), medication adherence is given double weight and lifestyle single weight.^c^

^a^ Good control requires positive outcomes in all factors; mixed outcomes indicate moderate control; poor outcomes across all factors indicate poor control. ^b^ Presence of comorbidity negatively affect control. ^c^ For individuals with diabetes duration below the 5–10-year threshold, medication adherence is weighted more heavily than lifestyle behaviours

##### Parameter sensitivity analysis

We performed a probabilistic sensitivity analysis to assess parameter uncertainty (Table 2, a detailed list of parameters and parameter-specific ranges and distributions in the published model description). Population parameters fitted to empirical data were excluded from the sensitivity analysis because their close alignment with observed patterns in empirical data (see the model description) makes varying them unlikely to yield meaningful insights. We generated 1000 parameter sets using Latin Hypercube Sampling to efficiently explore the parameter space [70]. For each simulation, we examined three key outcome measures: poor glucose control, poor medication adherence, and outpatient service utilisation.

To identify influential parameters, we calculated partial rank correlation coefficients (PRCC) using R’s sensitivity package. This approach measures the strength and direction of the relationship between each parameter and the outcomes while controlling for all other parameters. The resulting coefficients range from −1 to 1, with values closer to extremes indicating stronger associations. We considered parameters statistically significant at p < 0.05 to be influential for each outcome.

### Experiment design and scenarios

The model uses weekly timesteps to capture regular patient-provider interactions and medicine-taking behaviours, running for 10 years to capture both immediate behavioural responses and longer-term health outcomes, aligning with typical policy horizons [71]. Using the calibrated model with initial values set to 2024 trends, multiple simulations of current practice (Inter0) were run to establish baseline patterns of blood glucose control, medicine adherence, and healthcare utilisation; then four scenarios were run, each modifying specific model parameters while maintaining other baseline values:

1. Medicine availability scenario (Inter1): 10% increase in availability, making medicine more available in rural and urban areas.
2. Insurance coverage scenario (Inter2): 10% annual increase in NHIS active coverage for five consecutive years.
3. Sugar-sweetened beverage tax (Inter3): 20% tax affecting lifestyle parameters through obesity/overweight trends
4. Combined scenario (Inter 4): implementing both insurance expansion and sugar-sweetened beverage tax (Inter2 and Inter3).

**Fig. 3:**
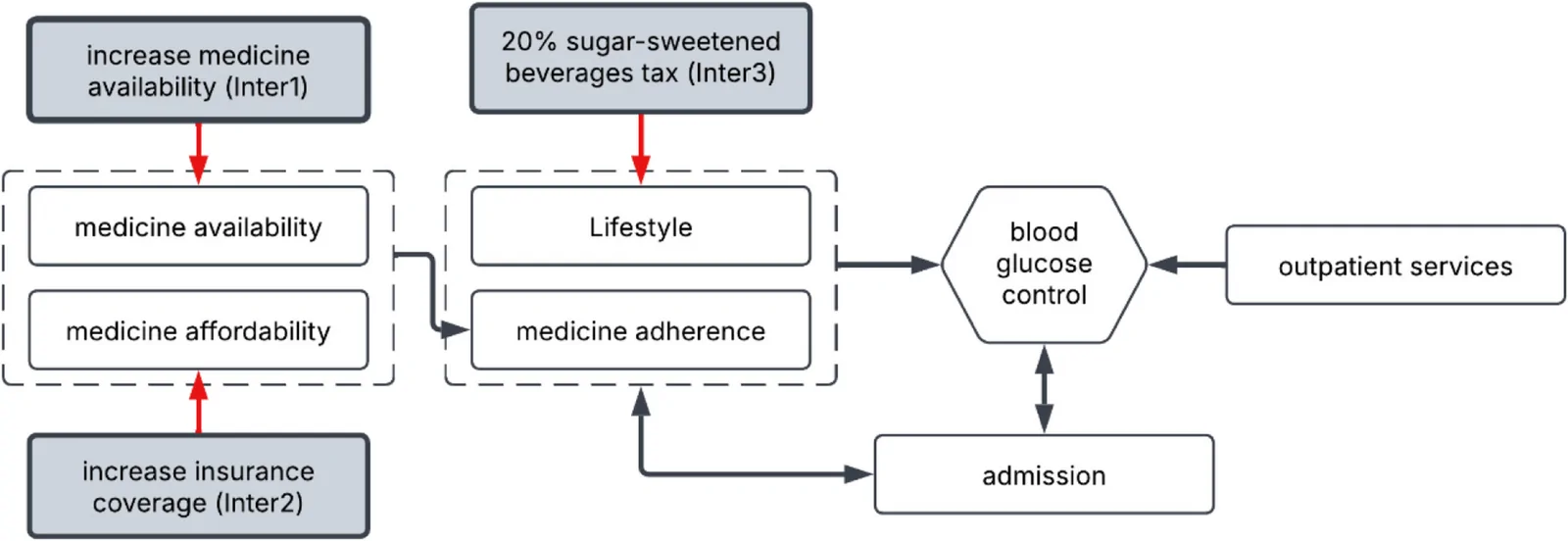
Pathway of scenario effect on diabetes treatment outcomes. Interventions (shaded boxes, solid red arrows) affect blood glucose control via medicine adherence and lifestyle. Providers support adherence and directly impact blood glucose control in admissions and outpatient care, while blood glucose control influences admission decisions.

These scenarios were selected based on the WHO Global Diabetes Targets 2022, the World Obesity Target, Ghana’s recent SSB policy initiatives and consultations with NHIS officials. While some scenarios (e.g., expanding NHIS coverage by 10% annually or increasing medicine availability by 10%) may not be immediately feasible, they are included to illustrate the potential impact of scaled interventions, providing insights into the magnitude of benefits that could be expected from more incremental, realistic strategies. Figure 3 shows how each scenario influences blood glucose control through different pathways.

For each scenario, changes in blood glucose control (good, moderate, and poor), medicine adherence, and healthcare utilisation are measured relative to the baseline.

### Simulation analysis

Scenario effects were evaluated by comparing outcomes to the baseline, estimating the difference in mean outcomes over a ten-year simulation period. Convergence was reached after 465 runs (± 5 incidences of good blood glucose control); therefore, we conducted 500 runs per experiment and 1000 for sensitivity analyses.

Key outcomes measured were blood glucose control (good/moderate/poor), medicine adherence (good: ≥ 80%, moderate: 50%−79%, poor: < 50%), and healthcare utilisation (admissions and outpatient visits). Blood glucose control and adherence were averaged over 13 weeks. Admissions were tracked weekly and accumulated over time.

The model was built in NetLogo 6.3 and analysed in R 4.2.2.

## Results

### Model fitting and confidence building

#### Population trend fitting results

The sigmoid function achieved over 80% predictive accuracy for fundamental population-level trends. The published model description shows the observed and predicted population trends. Predicted prevalence deviated from observed values by ~ 5%, with mean absolute predictive error 5.06% for lifestyle trends, 4.05% for insurance coverage, and 0.88% for comorbidity prevalence. These fitted trends provided the input data for subsequent analysis. The sigmoidal function effectively captured these increasing trends, with fitted parameter values provided in the published model description.

#### Population-level pattern analysis

Our model generates outcomes consistent with empirical studies across key population-level measures (Table 4). For blood glucose control, our baseline prediction of 35% good control (95% CI 34.1–35.2%) falls between values reported in the Ghanaian healthcare setting. The lower rate of 30% reported by Mobula et al. [64] comes from a broader facility study using a stricter control threshold (HbA1c < 7.0%), while the higher rate of 59% from Djonor et al. [5] reflects tertiary centre outcomes using a more lenient threshold (HbA1c < 8.0%). Similarly, our predicted good medication adherence rate of 72% (95% CI: 70.7–72.2%) falls within the range of 59%−83% found in meta-analyses from LMIC settings. The model focuses on the dynamics of blood glucose control and represents health facilities across Ghana that provide diabetes care. Its outcomes reflect patterns observed in the broader population.

**Table 4:** Comparing model outcomes to empirical data in published studies

Empirical data	Population summary	Reference	Model estimates proportion (95% CI)
*Good blood glucose control*			
0.300	1226 patients enrolled at five health facilities in Ghana (all levels included), with an average age of 57 ± 12.1 years. Controlled diabetes is defined as HbA1c < 7.0%	[63]	0.346 (0.341 – 0.352)
0.594 95% CI: 0.536—0.653	271 patients from a tertiary-level hospital in Ghana aged 56.6 ± 3.8 years. Controlled diabetes is defined as HbA1c < 8.0	[5]	
*Medicine adherence*			
median = 0.71 IQR = 0.59 – 0.83	19 studies from LMICs, in a meta-analysis, adherence measure included the mean number of days patients took medicines, MMAS-8 scale	[63]	0.715 (0.707—0.722)
*non-adherence to medications 0.434 95% CI: 0.175 – 0.694	13 studies in LMICs were used in the meta-analysis. Used the eight-item Morisky Medication Adherence Scale (MMAS-8) with a cut-off point of 80%	[64]	0.285 (0.277—0.292)

The model estimate is the proportion of patients in the outcomes under consideration at week 52 (1 year); it does not average the proportions across the 52 weeks (about 12 months) but rather the outcome on the 52nd week. The same applies to the proportion of patients who are adherent to medication. * The study reports nonadherence; if the result is expressed as the proportion adherent, we get 56.6%, like the model's estimate

#### Individual-level behaviour analysis

Examination of individual behaviour responses through extreme scenario testing showed clear patterns in how system constraints affect patient outcomes. Insurance coverage demonstrated substantially greater impact on medication adherence than medicine availability alone, with nearly 90% good adherence under full insurance compared to about 80% with full medicine access (see the published model description).

**Fig. 4:**
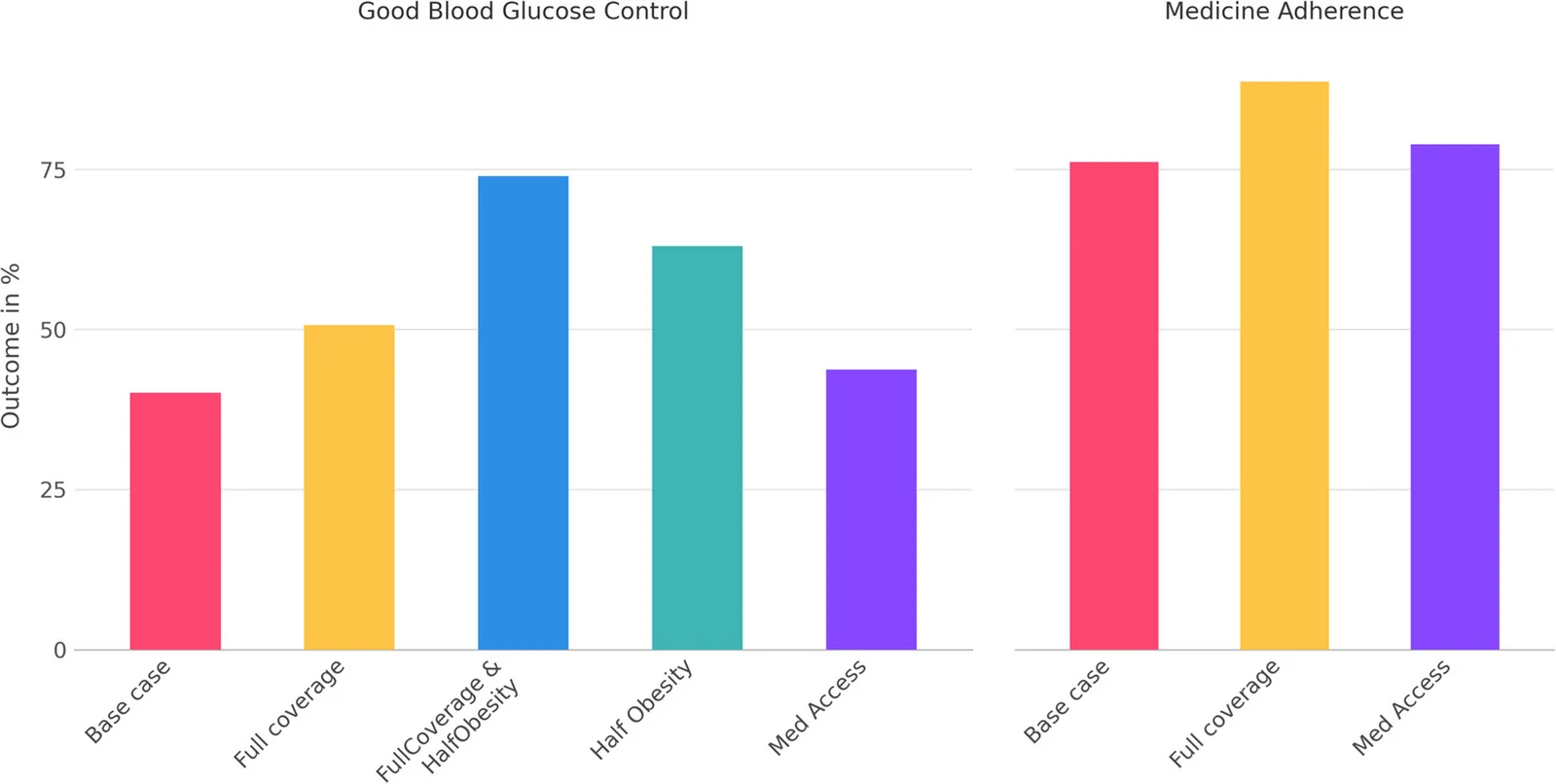
Blood glucose control and medicine adherence outcomes in structural sensitivity analysis. Base case = base case, the percentage of times patients found medicines in urban and rural communities was 80% and 70%, respectively. Insurance coverage in the base case was ~58% at initialisation. Full coverage = 100% insurance coverage. Half Obesity = half the prevalence of unhealthy lifestyle in rural and urban areas. Med Access = 100% access to medicines in rural and urban areas. FullCoverage & HalfObesity = a combined 100% insurance coverage and half the prevalence of unhealthy lifestyle in rural and urban areas. Lifestyle factors like obesity were excluded from the medication adherence scenarios, resulting in only three scenarios.

Similar patterns emerged for blood glucose control (Fig. 4). Analysis of relative scenario impacts showed that 100% insurance coverage led to a higher percentage of good blood glucose control (55%, 95% CI: 47%−53%) compared to scenarios with full medicine availability (40%, 95% CI: 38%−42%). Reducing obesity prevalence by half showed stronger effects (50% good control, 95% CI: 48%−52%) than increasing medicine availability alone.

Service providers confirmed that the patterns aligned with their clinical experience. In a structured discussion of scenario results, providers consistently identified affordability as the dominant barrier to medication adherence, noting that while stockouts influence adherence, patients can often locate medicines in private pharmacies but are prevented from accessing them due to cost. Recognising that prices in private pharmacies are likely higher than in public facilities without insurance, and that price sensitivity may not be linear, we interpret providers’ emphasis on out-of-pocket medicine expenses—whether in private or public settings—as a major barrier in the absence of insurance coverage. They also confirmed that lifestyle modifications effectively control blood glucose in the early T2DM, supporting the model’s prediction of strong impacts from obesity reduction (Fig. 4).

#### Treatment process analysis results

Service providers reviewed and confirmed key treatment process patterns in the model. Through structured assessment of example cases that included graphical representation of model outcomes, providers validated that the model appropriately captures how prescription renewals link to appointment attendance and how medicine costs affect adherence patterns. They confirmed these representations align with typical clinical practice in Ghana’s public health system.

#### Sensitivity analysis results

##### Structural sensitivity analysis

**Fig. 5:**
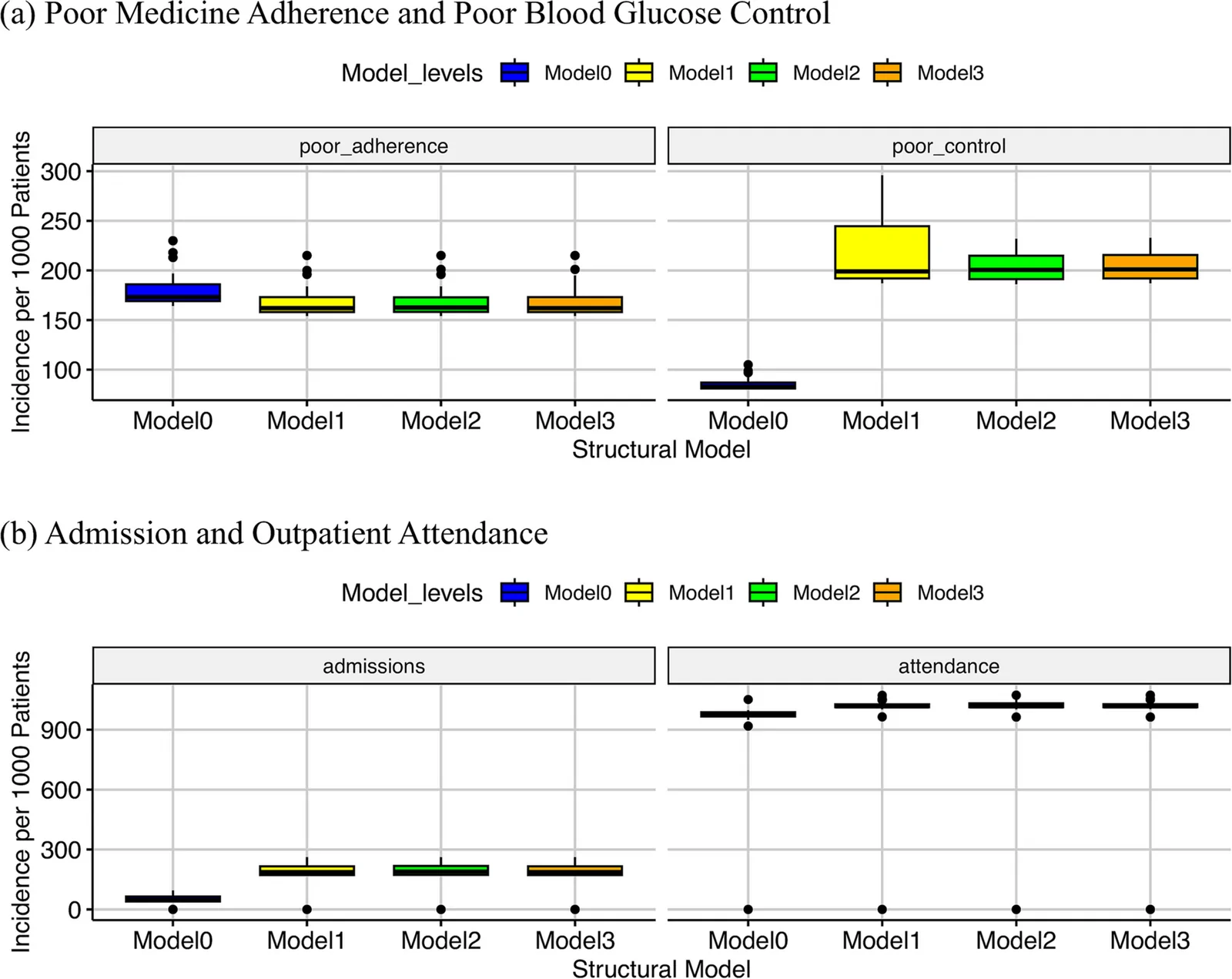
Outcomes of Structural Sensitivity Analysis on Models for Estimating Blood Glucose Control. Model0 represented the base case model, which estimated blood glucose control from medicine adherence and lifestyle. Model1 uses the weekly blood glucose control measure instead of a 3-month average to estimate control. Model2 estimates blood glucose control from medicine adherence, lifestyle and comorbidity. In Model3, the duration of diabetes is added to medicine adherence and lifestyle to estimate blood glucose control.

Structured sensitivity analysis demonstrated the robustness of our base model (Model 0) formulation for estimating blood glucose control. As shown in Fig. 5, while more complex formulations incorporating weekly measurement (Model1), comorbidity (Model2), and disease duration (Model 3) were tested, providers found the base case model better reflected observed clinical patterns. Panel (a) shows that the base case model predicts realistic rates of poor blood glucose control (about 10%), while more complex models overpredicted poor outcomes. Panel (b) demonstrates how these alternative formulations would overpredict healthcare utilisation, which is inconsistent with what providers observe.

##### Parameter sensitivity analysis

Parameter sensitivity analysis highlighted key influential factors (Table 5). Strong correlation between outpatient attendance (AttendanceProp) and outcomes (PRCC = −0.78, p < 0.001) aligns with the clinical understanding about the importance of regular healthcare contact. This is again confirmed by a moderate effect of the interval between review appointments (ReviewInterval) on outcomes. Regional variations in unhealthy lifestyle patterns showed moderate effects on blood glucose control (PRCC = 0.26–0.43). Results for all parameters included in the sensitivity analysis are in Supplementary File 2.

**Table 5:** Outcomes of significant parameters in probabilistic sensitivity analysis

Parameters	PRCC	P.value	95%CI lower	95%CI upper
*Poor blood glucose control*				
UrbanProDiet (unhealthy lifestyle in urban areas)	0.2586	0.0118	0.0595	0.5011
ruralProDiet (unhealthy lifestyle in rural areas)	0.4274	0	0.2448	0.6119
AttendanceProp	**−0.7841**	0	−0.8894	−0.7097
ReviewInterval	0.3256	0.001	0.1323	0.5448
*Poor medicine adherence*				
ruralProDiet (unhealthy lifestyle in rural areas)	−0.1945	0.0539	−0.4549	−0.011
AttendanceProp	**−0.8078**	0	−0.8866	−0.7416
ReviewInterval	0.3237	0.0038	0.1096	0.5141
*Incidence of admissions*				
AttendanceProp	**−0.8078**	0	−0.8866	−0.7416
ReviewInterval	0.3237	0.0038	0.1096	0.5141

*PRCC* Partial ranked correlation coefficient. 95% confidence intervals of the PRCC are presented. AttendanceProp = the proportion of outpatients attending scheduled review appointments, ReviewInterval = the time interval between outpatient review appointments

### Analysis of current patterns (Baseline)

#### System performance

Under baseline conditions (Inter0), our model shows moderate success in diabetes management, with 31.3% of patients achieving good blood glucose control, while 19.7% remained in poor control.

**Fig. 6:**
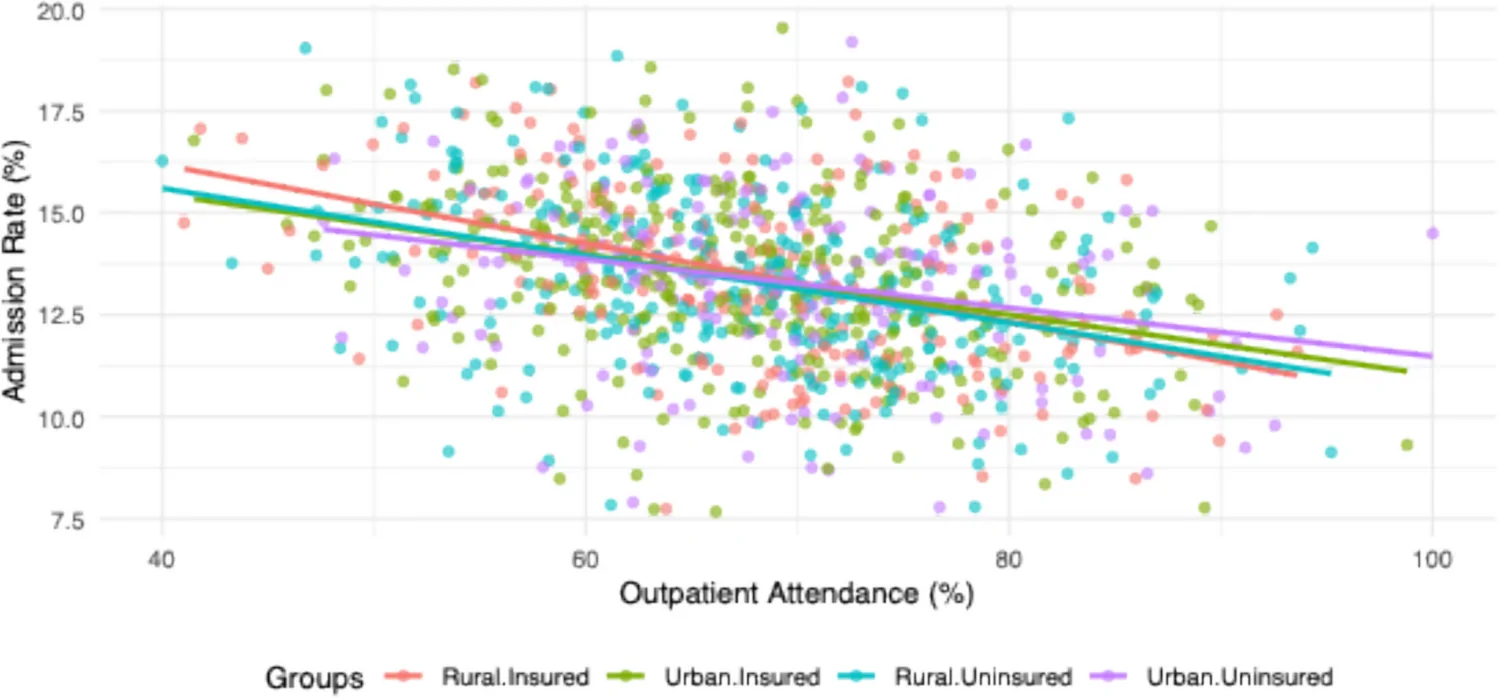
Admission and outpatient attendance by location and insurance coverage. Values plotted represent mean outcomes of 1000 simulations, each simulating 5 years. Trend lines indicate the best fit for the observed outcomes.

Medication adherence patterns revealed good outcomes, with 70% achieving good adherence. Service utilisation analysis revealed a relationship between outpatient care and hospital admissions (Fig. 6). On average, 10% of the population required hospital admission and 68% utilised outpatient services yearly. Admission rates decreased as outpatient visit frequency increased, suggesting the preventive benefits of regular outpatient care.

#### Health disparities

Our analysis reveals substantial disparities in outcomes based on both location and insurance status. Rural patients consistently experienced poor outcomes relative to urban patients, with 12% higher rates of poor blood glucose control and 44% higher rates of poor medicine adherence. These disparities stem from both reduced medicine availability in rural areas (60% vs 70% in urban areas) and lower insurance coverage rates (54% vs 62% in urban areas). When examining the interaction between location and insurance status, we found that insurance coverage substantially improves outcomes in both settings, with different magnitudes. Rural insured patients had a 1.32 (95% CI: 1.11–1.45) and 1.13 (95% CI: 1.03—1.23) times higher rate of good blood glucose control and good medicine adherence outcomes compared to rural uninsured patients. In urban areas, this effect was stronger, with insured patients achieving 1.70 (95% CI: 1.51 – 1.89) and 1.28 (95% CI: 1.02 – 1.54) times higher rates of good blood glucose control and good medicine adherence compared to the uninsured.

### Scenario impacts

#### Direct health outcomes

**Fig. 7:**
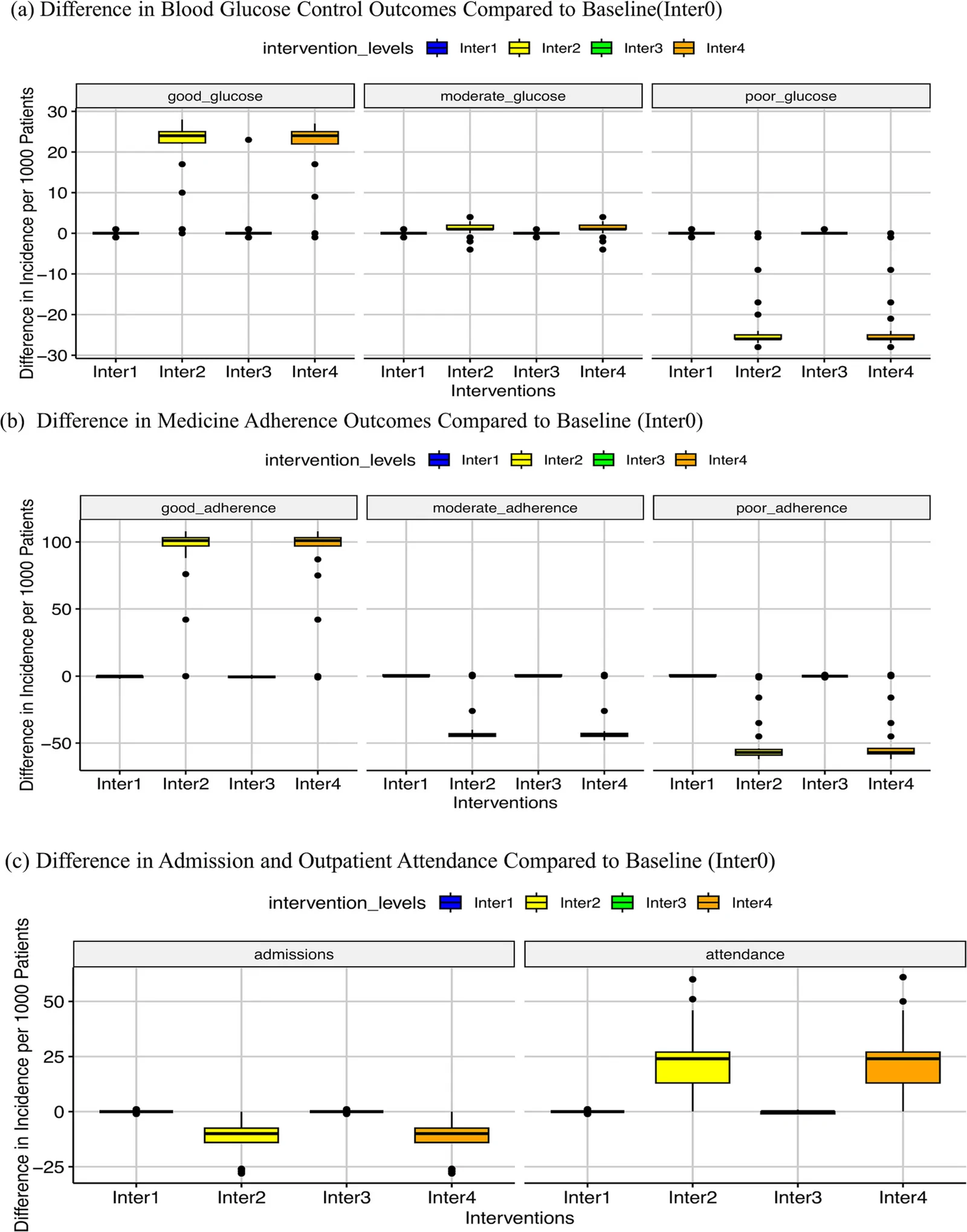
Direct health outcomes by interventions. Box plot of the outcomes after ten years under intervention scenarios. Inter0 = baseline, Inter1 = 10% increase in the percentage of times patients found medicines in rural and urban areas; Inter2 = 10% annual increase in the proportion of patients actively insured under the National Health Insurance Scheme for five consecutive years; Inter3 = 20% tax on sugar-sweetened beverages; Inter4 = combined Inter2 and Inter3. Good = incidence of good blood glucose control/ medicine adherence; moderate = incidence of moderately controlled blood glucose/ medicine adherence; poor = incidence of poorly controlled blood glucose/ medicine adherence. Lower hinge: 25% quantile; lower whisker: smallest observation greater than or equal to lower hinge − 1.5×IQR; middle: median; upper hinge: 75% quantile; upper whisker: largest observation less than or equal to upper hinge + 1.5×IQR; black dot: outlier.

Our scenario analysis showed substantial differences in effectiveness across the four scenarios tested (Fig. 7). Expanding insurance coverage (Inter2) demonstrated the strongest impact on blood glucose control compared to increasing medicine availability (Inter1) or implementing SSB tax (Inter3). The combined insurance coverage and SSB tax scenario (Inter4) showed similar magnitude effects as Inter2, primarily driven by the insurance component. Inter2 led to a significant decrease in poor control (mean of difference: 2.4%, 95% CI: 2.2 – 2.6) and an increase in good control (mean of difference: 2.2%; 95% CI: 2.1 – 2.4) compared to baseline (Inter0). In contrast, Inter1 and Inter3 showed no statistically significant improvements in blood glucose outcomes relative to baseline.

Effects on medication adherence followed a similar pattern but with larger magnitudes, as they are a conduit for the effect on control (Fig. 7b). The combined scenario (Inter4) led to a substantial increase in the incidence of good adherence (mean of difference: 9.4%, 95% CI: 8.6 – 10.1) and a decrease in the incidence of poor adherence (mean of difference: 5.2%, 95%: 4.8 – 5.7) compared to Inter0 after 10 years. Inter1 and Inter3 did not reduce the incidence of poor medicine adherence outcomes in Inter0.

#### Service delivery effects

Beyond clinical outcomes, our model revealed service utilisation shifts under different scenarios (Fig. 7c). Insurance expansion (Inter2) significantly reduced hospital admissions (1.4%, 95% CI: 1.1 – 1.6) while increasing outpatient visits (2.3%, 95% CI: 2.0 – 2.7) compared to Inter0. This pattern suggests a shift from acute to preventive care under expanded insurance coverage. Inter3 and Inter1 were no more effective at reducing admissions than Inter0.

#### Equity implications

Rural–urban disparities in health outcomes persisted after expanding insurance coverage. Rural patients continue to experience 15% lower rates of good blood glucose control and 22% lower rates of medicine adherence compared to urban patients, indicating the scenario does not effectively mitigate geographic inequities. Differential effects by baseline status revealed that expanding insurance coverage increases outpatient service utilisation (28% rise among rural patients and a 34% rise among urban patients) and improves blood glucose control by 1.38 times (95% CI: 1.21–1.51) in rural insured patients and 1.55 times (95% CI: 1.41–1.72) in urban insured patients. Similarly, medicine adherence increases by 1.19 times (95% CI: 1.08–1.31) in rural areas and 1.30 times (95% CI: 1.20–1.41) in urban areas. However, these gains do not sufficiently reduce the disparity in outcomes.

## Discussion

### Key findings and policy implications

This study aimed to improve understanding of how self-management behaviours interact with healthcare system constraints to influence T2DM outcomes in Ghana, and to evaluate the comparative effectiveness of policy scenarios in improving these outcomes. Three key findings of our analysis include: (1) financial barriers have a stronger impact on treatment outcomes than medicine availability constraints in this setting, with insurance expansion yielding substantially greater improvements than other scenarios; (2) significant disparities exist between rural/urban and insured/uninsured populations, with these disparities persisting even after insurance expansion; and (3) increasing outpatient service utilisation reduces hospital admissions highlighting the importance of regular monitoring in diabetes control. These findings demonstrate how affordability and availability barriers interact differently across population subgroups, with important implications for policy prioritisation in resource-constrained settings.

Our analysis highlights how various barriers to diabetes management interact within Ghana's public health system. While prescription renewal and medicine availability affect adherence and blood glucose control, financial barriers exert an even stronger impact. The interaction between insurance coverage and location-based disparities further reinforces this conclusion. Although both urban residence and insurance coverage improve physical and financial access to medicines, rural residence and lack of insurance are associated with limited medicine availability and greater financial challenges.

These findings corroborate previous empirical studies from Ghana [15, 58] and Nigeria [62], where insured hypertension patients had 4.5 times higher odds of medication adherence compared to the uninsured. In Ghana, insurance coverage rates are significantly higher for urban residents and those in high-income quintiles compared to rural and lower-income counterparts. Insurance renewal costs could explain why coverage remains lower in rural areas where a significant proportion of residents are poor [60]. While insurance helps overcome financial barriers in both settings, widely unavailable medicines in rural areas may limit the full benefit of insurance coverage, contributing to better health outcomes among urban insured populations relative to others.

Our results show that increasing outpatient service utilisation reduces the incidence of admissions, reinforcing the need for regular patient-provider interactions for effective management of diabetes and other chronic diseases. In our model, outpatient services include prescription renewal and temporarily controlling glucose in high-risk patients, which contribute to improved glucose control and reduce admission incidence. The finding corroborates evidence from Ghana [57] and international treatment guidelines on the value of patient-doctor interactions for effective diabetes management [10]. Regular contact with service providers offers opportunities to encourage and reinforce healthy self-management behaviours and to intervene to prevent potential complications that could lead to admission.

While our findings suggest expanding insurance coverage and reducing local-based disparities, the sustainability of Ghana’s NHIS must be considered. The scheme already faces financial challenges with current coverage levels, with delays in provider reimbursement and moral hazards among insured straining the system [72]. Expanding coverage, as we examined, would require additional funding to purchase clinical services for patients. An evaluation of the relative costs of this, reduction in hospital admissions leading to reduced costs, and reduction in long-term complications due to better glucose control would be valuable for policy decision-making and intervention design targeted at improving the disease control and NHIS sustainability. Future research should include economic analysis to determine whether the health benefits of expanded insurance coverage justify the additional investment, and to identify sustainable financing mechanisms that could support this expansion without compromising the scheme's long-term viability.

### Methodological contributions

Our agent-based approach extends healthcare systems modelling by integrating behaviour theories with system constraints to examine policy impacts. While insurance improved outcomes in both settings, its effect was stronger in urban areas (55% in urban vs 38% in rural), suggesting that physical access barriers limit the benefit of financial protection in rural areas. This study demonstrates the value of agent-based modelling for understanding healthcare access in resource-constrained settings. By modelling individual patient decisions within system constraints, we could examine how barriers compound to affect outcomes, an interaction difficult to study through traditional statistical approaches.

The pattern-oriented modelling approach we employed addressed data limitations common in resource-constrained healthcare settings. First, it helped develop confidence in the model with limited empirical data by using multiple complementary patterns at different levels [69]. Second, it provided a structured way to integrate diverse data sources, from clinical studies to service provider expertise—creating a more robust foundation for policy analysis. Third, it helped test and refine model assumptions by systematically assessing the model’s ability to reproduce multiple patterns, allowing us to identify which behavioural mechanisms were necessary for reproducing observed patterns. This multi-level validation approach proved particularly valuable for a context where longitudinal patient data was unavailable, but there were different cross-sectional patterns identified from different data sources.

Integrating behavioural theories into quantitative models offers a promising approach for examining self-management behaviours. Our model operationalised theoretical constructs from HBM and TPB, demonstrating how perceived barriers (like medicine cost) and behavioural control (like medicine availability) affect treatment outcomes. This integration helps bridge conceptual behavioural frameworks with quantitative healthcare systems models. For example, our model captures the psychological/internal processes of each patient using TPB’s attitude construct, while social and environmental barriers are represented using insights from subjective norms and actual behavioural control constructs.

The multi-level modelling capability of ABM proved valuable for quantifying how system constraints affect healthcare outcomes through patient behaviours. This ability arises from ABM’s capacity to represent heterogeneous behaviours and complex social networks, and to simulate interactions between individual-level decisions and system-level constraints [44]. By explicitly modelling these cross-level interactions, we could examine how policy scenarios targeting system constraints (like medicine availability) translate to behavioural changes (like adherence) and ultimately impact clinical outcomes (like blood glucose control), providing a more complete picture of scenario pathways and bottlenecks.

## Limitations and conclusion

While our ABM improves understanding of self-management behaviour and scenario effects, it has limitations. Our model captures key factors but not all potentially relevant covariates of blood glucose control, such as other healthcare services (e.g., hypertension, obesity), factors influencing medicine nonadherence (e.g., stress, forgetfulness), use of non-orthodox medicines (e.g., herbs) and broader contextual factors like urbanisation, transportation, and service availability that may impact self-management and blood glucose control. Expanding the model to include these factors could improve its predictive power. We conducted uncertainty analysis on the structural models for blood glucose estimation, but the complexity of blood glucose regulation in the individual body could limit the generalisability of our findings.

A second limitation concerns our data sources and validation approach. Despite being the first to examine the impact of SSB tax on diabetes control in Ghana, we used effect estimates from India and South Africa, which may not reflect the effects in Ghana. Additionally, we did not model the details of how the SSB tax reduces consumption and promotes a healthy body mass index, leading to improvement in blood glucose control. Quality data on policy effects would enhance model parameters. Moreover, our analysis indicates that patients’ diet and exercise habits, the frequency of outpatient appointment attendance, and the intervals between appointments have a significant influence on blood glucose control. Consequently, high-quality, granular data on these parameters need to be collected. Further studies are required to validate assumptions surrounding behaviour theories incorporated in the model, particularly the assumption that intentions lead to behaviour in the application of HBM and TPB.

In conclusion, this work demonstrates how agent-based modelling can reveal the complex interactions between individual behaviours and system constraints in resource-limited settings. By capturing these interactions, we show how policy scenarios must account for both individual barriers and system capacity to effectively improve healthcare access for underserved populations. Our findings highlight the importance of prioritising insurance expansion over medicine availability or SSB taxation while also recognising that addressing financial barriers alone will not eliminate rural–urban disparities in diabetes outcomes. The methodology developed here provides a framework for analysing similar complex health system challenges where data is limited but pattern-oriented validation is possible.

## Supplementary Information

Below is the link to the electronic supplementary material.

Supplementary file1 (DOCX 23 KB)

Supplementary file2 (DOCX 27 KB)

Supplementary file3 (DOCX 31 KB)
