# Supplementary material for: Improving treatment outcomes in Ghana with agent-based model for diabetes patients’ self-management behaviours
Source: Health Care Manag Sci. 2026 Jul 25;29(3):35. doi: 10.1007/s10729-026-09772-8 (PMC13401566; doi:10.1007/s10729-026-09772-8)
Supplement: Supplementary file 1 — Supplementary file1 (DOCX 23 KB) [file 10729_2026_9772_MOESM1_ESM.docx]

Improving treatment outcomes in Ghana with Agent-based model for diabetes patients’ self-management behaviours

Eunice Twumwaa Tagoe^1*^ [ORCID: <https://orcid.org/0000-0002-2198-4980> ]

Justice Nonvignon^2^ [ORCID: https: <https://orcid.org/0000-0002-7484-9491>]

Robert Van Der Meer^3^ [ORCID: <https://orcid.org/0000-0002-9442-1628>]

Itamar Megiddo^3^ [ORCID: <https://orcid.org/0000-0001-8391-6660>]

^1^Population Health Sciences Institute, Newcastle University, Newcastle upon Tyne, UK

^2^School of Public Health, University of Ghana, Accra, Ghana

^3^Department of Management Science, University of Strathclyde, Glasgow UK

*Correspondence: Eunice Twumwaa Tagoe. Email: eunice.adwubi@newcastle.ac.uk

Journal Name: Healthcare Management Science

Supplementary File 1: Summaries of Model Patterns and Findings from Health Service Provider Engagement

The document summarises insights from policy documents, semi-structured interviews with clinicians and informal discussions on diabetes management in Ghana and how the insights are incorporated into the model. Details of the semi-structured interviews is published here: <https://doi.org/10.1177/13558196221111708>

**Purpose and methods:**

This appendix documents how healthcare provider expertise informed model development and validation through a pattern-oriented modelling approach.

We engaged with clinicians through both published semi-structured interviews (Tagoe et al., 2023) and a series of formal discussions to identify and validate patterns related to medicine adherence and blood glucose control to ensure that our model reproduces patterns observed in reality.

Three physicians (with 2+ years of experience) were engaged in a series of discussions to validate the model. They were purposively selected from interviewees involved in the qualitative study (details in Tagoe et al., 2023). There were three online group discussions. Providers were asked series of open-ended questions and presented with examples of patient case profiles from the model, scenarios and their outcomes in graphical format, to discuss.

To minimise confirmation bias, we solicited provider feedback before showing model outputs, and structured questions to avoid suggesting expected answers. All discussions were documented through recording and noting taking. There were no disagreements between providers.

**Key patterns Identified and Model Adaptation**

***Patterns identified from the summary of interviews and the Ghana National Health Insurance Scheme operations manual.***

1. Outpatient appointments are scheduled considering availability and the mode of payment. Even when available, appointments in insured patients are capped at six times in 12 months, according to the National Health Insurance Scheme (NHIS) operational manual. According to clinicians, patients who miss outpatient appointments are not rescheduled; they attend appointments later.

We modelled insured patients to have a maximum of 6 appointments in 12 months. Patients who missed outpatient appointments set their next appointment to any random week between the time they missed an appointment and the interval between outpatients' review appointments (i.e., 13 weeks).

2. Admissions are usually based on blood glucose control and comorbidity status. Clinicians explained that poorly controlled glucose in patients with hypertension and cardiovascular disease conditions is admitted, allowing for adequate care. Clinicians explained that poorly controlled blood glucose in patients with no comorbidity is usually controlled in hospitals in less than 24 hours but not admitted.

We modelled admissions in patients with poorly controlled glucose and comorbidity. Patients without comorbidity but poorly controlled glucose who attend health facilities have their glucose controlled.

3. The interval between outpatients' review appointments is mainly based on blood glucose control and comorbidity status. Clinicians schedule patients who have controlled diabetes and no concerns with comorbidities for 13 weeks. Clinicians consider the availability of appointments and do not exceed six outpatient appointments in insured patients.

We used a 13-week interval between outpatient appointments in the model.

***Patterns identified from informal interactions with clinicians.***

The service providers raised specific issues that were not addressed in the initial model:

1. Clinicians considered our initial assumption that patients who do not find medicines to buy in the week they received prescriptions do not take them until their next appointment unrealistic. They pointed out that patients often search for medicines in community outlets and may obtain them before their next appointment. Additionally, prescriptions are typically valid until the next appointment, so once patients purchase the medicines, they do not search for them again until after the next prescription.

In response, we updated the model to allow patients to search for medicine every week until they have medicines. During periods when patients do not attend appointments as scheduled, we assumed patients stopped taking medicines since the prescription would have finished.

2. Clinicians noted that the assumption that increasing hospital admissions leads to fewer outpatient appointments in the preceding weeks is rarely the case. They explained that most patients with severely uncontrolled blood sugar levels are kept in the hospital for a week or two to bring their blood glucose under control and are then sent home. Only a small proportion of patients (1 out of 10) are admitted. As a result, there are few admissions each week, which does not significantly affect the number of outpatient appointments.

In response, we removed the assumption from the model. Thus, the number of admissions did not reduce the number of outpatient appointments scheduled for a week.

3. We modelled discharged patients to follow lifestyle and medicine adherence behaviours before admission. Clinicians observed that discharged patients adhered to medicines and lifestyle recommendations after discharge for fear of readmission. However, these behaviours are short-lived because clinicians are not present to motivate and monitor patients' behaviour post-discharge. According to clinicians, lifestyle behaviours are formed early in life and do not change significantly after an adverse event; however, long-term intervention could help improve diet behaviours.

In response, we modelled discharged patients to engage in healthy lifestyles and take medicine (if available and affordable) until after their review appointment preceding discharge.

4. Concerning the schedule of activities, we originally estimated weekly blood glucose control from lifestyle and medicine adherence behaviour. Clinicians suggested that estimating blood glucose control weekly could be misleading. They recommended using an overall control over time, like HbA1c, as a more accurate representation of blood glucose, as it represents the most occurring control over a 3–4-month period.

In response, we estimated the average blood glucose control over three months (13 weeks) by collecting weekly blood glucose data from patients. We calculated the 13-week average as the most frequently occurring state a patient experienced. In the case of a tie, the patient was randomly allocated to one of the states.

5. Clinicians emphasised that medicines and diet are the main predictors of blood glucose in diabetes patients, and the relative importance of these predictors can vary based on the duration of diabetes. Diet is particularly effective in controlling glucose in the early stages of the disease (0 – ~5 years). While the impact of diet on blood glucose control decreases with ageing and physiological changes, medicine is assumed to contribute significantly to control.

In response, we maintained an equal weighting for lifestyle and medicine adherence in the baseline. We explored the concept of ageing and the duration of diabetes in a structural uncertainty analysis.

6. We originally arranged the model operations so that blood glucose is estimated, followed by lifestyle and medicine adherence behaviour. Clinicians recommended rearrangement of the order of operations in the model. According to them, blood glucose control reflects patients' medication adherence and diet/exercise behaviour. Thus, patients must engage in these behaviours before blood glucose is estimated.

We adjusted the order of operations so that outpatients were assessed for lifestyle and medicine intake behaviours before their blood glucose state was estimated.

7. Initially, inpatients were set to follow their prior lifestyle. However, clinicians explained that inpatients are supervised to take medicines as prescribed and eat healthily.

Consequently, we adjusted the model to reflect clinicians' opinions; inpatients ate healthily and took medicines as prescribed.
