# Supplementary material for: Improving treatment outcomes in Ghana with agent-based model for diabetes patients’ self-management behaviours
Source: Health Care Manag Sci. 2026 Jul 25;29(3):35. doi: 10.1007/s10729-026-09772-8 (PMC13401566; doi:10.1007/s10729-026-09772-8)
Supplement: Supplementary file 2 — Supplementary file2 (DOCX 27 KB) [file 10729_2026_9772_MOESM2_ESM.docx]

Improving treatment outcomes in Ghana with Agent-based model for diabetes patients’ self-management behaviours

Eunice Twumwaa Tagoe^1*^ [ORCID: <https://orcid.org/0000-0002-2198-4980> ]

Justice Nonvignon^2^ [ORCID: https: <https://orcid.org/0000-0002-7484-9491>]

Robert Van Der Meer^3^ [ORCID: <https://orcid.org/0000-0002-9442-1628>]

Itamar Megiddo^3^ [ORCID: <https://orcid.org/0000-0001-8391-6660>]

^1^Population Health Sciences Institute, Newcastle University, Newcastle upon Tyne, UK

^2^School of Public Health, University of Ghana, Accra, Ghana

^3^Department of Management Science, University of Strathclyde, Glasgow UK

*Correspondence: Eunice Twumwaa Tagoe. Email: eunice.adwubi@newcastle.ac.uk

Journal Name: Healthcare Management Science

Supplementary File 2: Results of Probabilistic Sensitivity Analysis

The results represent outcomes in 1000 simulations, each using unique parameter sets drawn using Latin Hypercube Sampling to efficiently explore the parameter space.

|  | Parameters | PRCC | P.value | 95%CI - lower | 95%CI - upper |
| --- | --- | --- | --- | --- | --- |
| ***Good BGC*** | | | | | |
| **1** | MedRural | -0.1229 | 0.2648 | -0.3436 | 0.1355 |
| **2** | MedUrban | -0.0029 | 0.98 | -0.2798 | 0.2151 |
| **3** | UrbanProp | -0.0397 | 0.7068 | -0.2533 | 0.1795 |
| **4** | UrbanProDiet (unhealthy lifestyle in urban areas) | -0.7105 | 0 | -0.8184 | -0.615 |
| **5** | RuralProDiet | -0.6084 | 0 | -0.7409 | -0.4885 |
| **6** | AttendanceProp | 0.3849 | 0.0001 | 0.1893 | 0.5682 |
| **7** | ReviewInterval | -0.1852 | 0.0949 | -0.3996 | 0.0221 |
| **8** | LenStay | 0.0111 | 0.9253 | -0.2687 | 0.204 |
| ***Moderate BGC*** | | | | | |
| **1** | MedRural | 0.0778 | 0.4828 | -0.1754 | 0.2764 |
| **2** | MedUrban | -0.0132 | 0.9159 | -0.2621 | 0.2657 |
| **3** | UrbanProp | -0.107 | 0.3353 | -0.3581 | 0.1109 |
| **4** | UrbanProDiet | 0.5297 | 0 | 0.3623 | 0.7186 |
| **5** | RuralProDiet | 0.338 | 0.001 | 0.1604 | 0.6057 |
| **6** | AttendanceProp | 0.0764 | 0.5508 | -0.2025 | 0.3097 |
| **7** | ReviewInterval | 0.0357 | 0.7429 | -0.2171 | 0.2084 |
| **8** | LenStay | -0.0465 | 0.6684 | -0.2955 | 0.1284 |
| ***Poor BGC*** | | | | | |
| **1** | MedRural | -0.0283 | 0.8041 | -0.2744 | 0.2119 |
| **2** | MedUrban | 0.0437 | 0.6827 | -0.1543 | 0.2806 |
| **3** | UrbanProp | -0.0855 | 0.2972 | -0.248 | 0.0789 |
| **4** | UrbanProDiet | 0.2586 | 0.0118 | 0.0595 | 0.5011 |
| **5** | RuralProDiet | 0.4274 | 0 | 0.2448 | 0.6119 |
| **6** | AttendanceProp | -0.7841 | 0 | -0.8894 | -0.7097 |
| **7** | ReviewInterval | 0.3256 | 0.001 | 0.1323 | 0.5448 |
| **8** | LenStay | 0.0466 | 0.6808 | -0.1428 | 0.3274 |
| ***Good medicine adherence*** | | | | | |
| **1** | MedRural | 0.0707 | 0.4373 | -0.1136 | 0.2742 |
| **2** | MedUrban | -0.005 | 0.9672 | -0.2957 | 0.1917 |
| **3** | UrbanProp | -0.072 | 0.5713 | -0.3244 | 0.1716 |
| **4** | UrbanProDiet | 0.0643 | 0.4996 | -0.0875 | 0.2782 |
| **5** | RuralProDiet | 0.0651 | 0.5723 | -0.1715 | 0.3078 |
| **6** | AttendanceProp | 0.8335 | 0 | 0.7638 | 0.9147 |
| **7** | ReviewInterval | 0.0587 | 0.5495 | -0.1218 | 0.2894 |
| **8** | LenStay | 0.0301 | 0.8204 | -0.2228 | 0.3202 |
| ***Moderate medicine adherence*** | | | | | |
| **1** | MedRural | 0.0778 | 0.4816 | -0.1777 | 0.3014 |
| **2** | MedUrban | -0.0132 | 0.9061 | -0.2288 | 0.2411 |
| **3** | UrbanProp | -0.107 | 0.46 | -0.4271 | 0.1858 |
| **4** | UrbanProDiet | 0.5297 | 0 | 0.3768 | 0.712 |
| **5** | RuralProDiet | 0.338 | 0.0012 | 0.1062 | 0.5246 |
| **6** | AttendanceProp | 0.0764 | 0.5046 | -0.1281 | 0.2919 |
| **7** | ReviewInterval | 0.0357 | 0.7231 | -0.1621 | 0.2239 |
| **8** | LenStay | -0.0465 | 0.7122 | -0.2886 | 0.1979 |
| ***Poor medicine adherence*** | | | | | |
| **1** | MedRural | -0.0409 | 0.6721 | -0.2543 | 0.1596 |
| **2** | MedUrban | -0.0425 | 0.6589 | -0.2761 | 0.1283 |
| **3** | UrbanProp | -0.1143 | 0.2441 | -0.3154 | 0.0604 |
| **4** | UrbanProDiet | -0.1109 | 0.364 | -0.4023 | 0.159 |
| **5** | RuralProDiet | -0.1945 | 0.0539 | -0.4549 | -0.011 |
| **6** | AttendanceProp | -0.8078 | 0 | -0.8866 | -0.7416 |
| **7** | ReviewInterval | 0.3237 | 0.0038 | 0.1096 | 0.5141 |
| **8** | LenStay | -0.0664 | 0.5271 | -0.2723 | 0.1218 |
| ***Incidence of admissions*** | | | | | |
| **1** | MedRural | -0.1274 | 0.2691 | -0.3731 | 0.0913 |
| **2** | MedUrban | 0.0747 | 0.5183 | -0.2027 | 0.272 |
| **3** | UrbanProp | 0.0291 | 0.7845 | -0.2271 | 0.2566 |
| **4** | UrbanProDiet | 0.3614 | 0.0006 | 0.1391 | 0.5875 |
| **5** | RuralProDiet | 0.5597 | 0 | 0.4068 | 0.7128 |
| **6** | AttendanceProp | -0.8225 | 0 | -0.8942 | -0.7524 |
| **7** | ReviewInterval | -0.1497 | 0.1369 | -0.3688 | 0.0469 |
| **8** | LenStay | 0.0349 | 0.7531 | -0.1672 | 0.2415 |
| ***Outpatient Attendance*** | | | | | |
| **1** | MedRural | -0.0444 | 0.6478 | -0.2418 | 0.1885 |
| **2** | MedUrban | 0.0058 | 0.9616 | -0.2974 | 0.2263 |
| **3** | UrbanProp | -0.0905 | 0.4981 | -0.3515 | 0.193 |
| **4** | UrbanProDiet | 0.0744 | 0.4301 | -0.0847 | 0.2879 |
| **5** | RuralProDiet | 0.1325 | 0.2403 | -0.077 | 0.3752 |
| **6** | AttendanceProp | 0.732 | 0 | 0.6413 | 0.8535 |
| **7** | ReviewInterval | -0.4631 | 0 | -0.6455 | -0.2852 |
| **8** | LenStay | 0.0115 | 0.9328 | -0.2655 | 0.2686 |

Note: PRCC = partial rank correlation coefficients. The coefficients range from -1 to 1, with values closer to extremes indicating stronger association, negatives indicating negative association (increasing the parameter value reduces the impact on the outcome and vice versa) and positives indicating a positive association (increasing parameter values increases the impact on the outcome) MedRural = medicine availability in rural areas. MedUrban = medicine availability in urban areas. UrbanProp = the proportion of the modelled cohort residing in an urban area. UrbanProDiet = the prevalence of unhealthy lifestyle (proxied from obesity and overweight prevalence) in urban areas. RuralProDiet = the prevalence of unhealthy lifestyle (proxied from obesity and overweight prevalence) in rural areas. AttendanceProp = the proportion of outpatients attending scheduled review appointments, ReviewInterval = the time interval between outpatient review appointments. LenStay = the maximum number of weeks spent in admission.
