# Supplementary material for: Improving treatment outcomes in Ghana with agent-based model for diabetes patients’ self-management behaviours
Source: Health Care Manag Sci. 2026 Jul 25;29(3):35. doi: 10.1007/s10729-026-09772-8 (PMC13401566; doi:10.1007/s10729-026-09772-8)
Supplement: Supplementary file 3 — Supplementary file3 (DOCX 31 KB) [file 10729_2026_9772_MOESM3_ESM.docx]

Improving diabetes treatment outcomes: Agent-based modelling of patients’ self-management behaviours and treatment outcomes in Ghana

Eunice Twumwaa Tagoe^1*^ [ORCID: <https://orcid.org/0000-0002-2198-4980> ]

Justice Nonvignon^2^ [ORCID: https: <https://orcid.org/0000-0002-7484-9491>]

Robert Van Der Meer^3^ [ORCID: <https://orcid.org/0000-0002-9442-1628>]

Itamar Megiddo^3^ [ORCID: <https://orcid.org/0000-0001-8391-6660>]

^1^Population Health Sciences Institute, Newcastle University, Newcastle upon Tyne, UK

^2^School of Public Health, University of Ghana, Accra, Ghana

^3^Department of Management Science, University of Strathclyde, Glasgow UK

*Correspondence: Eunice Twumwaa Tagoe. Email: eunice.adwubi@newcastle.ac.uk

Journal Name: Healthcare Management Science

Supplementary File 3: Model verification

This document describes how the ABM conceptual model was tested against the implemented model.

To verify the model, we performed several activities. We employed a bottom-up testing approach, incrementally integrating sub-models from the simplest low-level components to the more complex dependent ones. We then traced randomly selected patients through the inspect function in the NetLogo interface. We identified code errors using the debugger function in NetLogo and fixed them. We employed a white-box testing approach to verify the model. White-box testing is a software testing methodology that involves testing the internal logic and structure of a model to ensure the application functions as intended [1]. We designed test cases with the expected outcomes determined before running them. We ran the test cases to confirm that the expected outcomes match the model outputs. Details of the test cases are described below. Table 1 contains pseudocode showing the implementation logic. After making changes to the model, we retested the set of cases in white-box testing to ensure the modifications did not introduce new errors or negatively affect the model's existing functionality.

*Cohort size*

1. At any point in the model, the count of inpatients, outpatients and discharged patients must equal the total number of patients. We coded the model to print errors whenever the condition is not met. We ran 100 iterations of the base case model to test, and no errors were reported.

*Medicine adherence and access*

2. In the conceptual model, we expected all outpatients (clinical-state = 2) who missed appointments (appointment > current week) to have no medicines or outstanding prescriptions to buy (prescription = -1). Also, we expected that patients who had no insurance (insurance = 0) and were very poor (wealth <= 1) could not afford medicines and thus would not check for medicine availability in their community (check-med-in-com). We asked patients checking medicine availability to print an “error” if they didn’t meet the conditions.

3. The number of patients who do not have medicines (prescription = -1) should be equal to or less than the number of patients who have missed appointments in a week. Otherwise, there is an error or bug in the code. Patients who do not have medicines constitute missed appointments, and outpatients who cannot afford them. We coded the model to print out an error message if the difference in missed appointments and patients with no meds exceeds zero.

*Diet behaviour per clinical-state*

4. We expected the prevalence of poor diet behaviour among outpatients, including those discharged, to match the predicted value of PDiet (a global variable in the model). We also expected all inpatients to have good diet behaviours and be medicine adherent. Consequently, we ran the model 100 times and printed an error message if the condition was not met.

*Hospital services*

5. We expected patients who attended health facilities due to emergencies or review appointments to be admitted if they had poorly controlled blood glucose. On the week of discharge, these patients should have a recovery period equal to the time until their next appointment. To verify, we traced, in turn, 50 randomly selected inpatients until their week of discharge and their next appointment, observing their clinical state, recovery period, and appointment. We confirmed that the recovery-period and appointment were equal to the current week (ticks), clinical-state = 2 (outpatient/recovered for the complication leading to admission).

6. Given that diet and medicine adherence influenced blood glucose control in the base case, we expected that if all patients adhered to medication and ate healthily, they would all have good control. Consequently, there should be no admissions. The opposite is expected if all patients ate unhealthily and did not take medicines. We confirmed the expected when we ran the model under these conditions.

Table 1 describes how the model procedures have been implemented to ensure alignment between their conceptualisation and their coding in NetLogo. See the model description document for a detailed description of conceptual elements and pseudocode describing implementation logic:

<https://doi.org/10.15129/e0d78b33-152f-44dc-9d8e-07886db72288>

Table 1: Model verification

| Conceptual element | Description | ABM implementation (pseudo code/implementation logic) |
| --- | --- | --- |
| check-clinical-state | The procedure checks if a discharged patient has fully recovered from an acute blood glucose complication that led to admission. It then updates such patients' ***clinical-state***, which influence subsequent procedures patients perform in the model. | At the start of each time step   - for discharged patients ( - if recovered (recovery_period = current week) - become an outpatient (set clinical_state = outpatient) - decide on lifestyle (set_diet) |
| Diet behaviour per clinical-state | All patients perform this procedure weekly (every time step) to update the weekly record (*my-lifestyle*) of their lifestyle. inpatients and discharged patients live healthily. All patients simply update their *my-lifestyle* list by appending the current *lifestyle* to the end of the list. The list is used to decide predominant lifestyle over 3 months to feed into blood glucose control estimation. | - for discharged and inpatients - set-diet healthy - for all patients - record my-lifestyle this week - add my lifestyle to my -lifestyle record |
| Medicine adherence and access: *check-meds* | Outpatients with prescription (*clinical-state* = 0 or *clinical-state* = 1) perform the procedure to decide medicine availability in their community and whether they can afford medicines. The procedure constitutes two submodels: ***check-affordability*** and ***check-med-in-com****,* in the order of execution. | - To check medicine availability: for outpatients and the discharged (clinical_state = outpatient or discharged) - If you have a valid prescription (prescription = valid) - Check if you can afford medicine even before looking to them (check-affordability) - If insured or in the middle to high-income quintile, you can afford otherwise you can’t - If can afford, check medicine availability, if can’t afford, check affordability next week - To check medicine availability, draw from a uniform distribution between 0-; compare draw to medicine availability parameter in rural or urban setting depending on place of residence. - If medicine is available purchase, if not check availability next week. |
| Medicine adherence and access: *take-med* | Describes patients medicine intake/adherence behaviours. Depending on *clinical state*, all patients perform some part of this procedure every week. Outpatients, including those discharged who have medicines at hand are set to take medicines. Outpatients without medicines do not take medicines but rather check accessibility. Inpatients take medicines. | - If inpatient, take medicine as prescribed - If outpatient or discharged with medicine at hand, take medicine, - If outpatient or discharged without medicine at hand but a valid prescription, - check affordability and availability - If outpatient or discharged without medicine at hand and no valid prescription, - do not take medicine |
| Health services | Describes patient decision to attend outpatient appointment and clinical services provided during such appointments. The procedure constitutes four submodels: ***decide-attendance, treat, admit*** and ***schedule-appointment*** | ***decide-attendance***  For outpatients with appointment in current week,  if poorly controlled blood glucose attend, otherwise  decide on attendance using stochasticity and review attendance parameter  for outpatients without appointment in current week,  if comorbid and poorly control blood glucose, attend (emergency)  ***treat***  If good or moderately controlled blood glucose, prescribe and ***schedule next appointment***,  Next appointment in good control between 9-13 weeks, based on availability  Next appointment in moderate control between 4-9 weeks, based on availability  Prescription to cover interval between appointment  If poorly controlled glucose  ***admit*** (for 1-2 weeks, drawn from a random uniform distribution)  Set clinical status to inpatient  Set discharge week  Healthy lifestyle  Medicine adherence  Restore glucose control  **discharge**  if discharge week is current week,  Set clinical state discharged  Next appointment between 1-4 weeks  Prescribe  Healthy lifestyle  Medicine adherence |

Reference

[1] Henard C, Papadakis M, Harman M, Jia Y, Le Traon Y. Comparing white-box and black-box test prioritization. Proceedings of the 38th International Conference on Software Engineering, Austin Texas: ACM; 2016, p. 523–34. https://doi.org/10.1145/2884781.2884791.
